# Detection of ADTRP in circulation and its role as a novel biomarker for coronary artery disease

**DOI:** 10.1371/journal.pone.0237074

**Published:** 2020-08-13

**Authors:** Delicia Shu Qin Ooi, Sze Min Ong, Ming Hui Eng, Yiong Huak Chan, Yung Seng Lee, Adrian Fatt Hoe Low, Mark Yan-Yee Chan, Chew-Kiat Heng

**Affiliations:** 1 Department of Paediatrics, Yong Loo Lin School of Medicine, National University of Singapore, Singapore, Singapore; 2 Khoo Teck Puat-National University Children’s Medical Institute, National University Health System, Singapore, Singapore; 3 Biostatistics Unit, Yong Loo Lin School Medicine, National University of Singapore, Singapore, Singapore; 4 Department of Medicine, Yong Loo Lin School of Medicine, National University of Singapore, Singapore, Singapore; 5 National University Heart Centre, National University Health System, Singapore, Singapore; University of Tennessee Health Science Center College of Medicine Memphis, UNITED STATES

## Abstract

Androgen dependent tissue factor pathway inhibitor regulating protein (ADTRP) is a novel protein associated with coronary artery disease (CAD) susceptibility, and reduced mRNA expression of ADTRP was shown to be associated with increased CAD risk. This study aimed to determine and compare circulating ADTRP levels between CAD patients and controls, and to test the performance of plasma ADTRP as a biomarker for CAD. We measured plasma ADTRP, tumor necrosis factor-alpha (TNF-α), interleukin-6 (IL-6) and high sensitivity-C reactive protein (hs-CRP) levels in 362 CAD patients, 150 angiographically negative CAD controls, and 83 healthy adults with no known clinical or medical conditions using commercial ELISA. Statistical analyses were performed using receiver operator characteristic (ROC) curves, quantile regression and logistic regression, with adjustments for age, gender, ethnicity and BMI. CAD patients had significantly lower plasma ADTRP levels 1,545 (1,087–2,408) pg/ml as compared to CAD controls 2,259 (1,533–3,778) pg/ml and healthy adults 3,904 (2,732–5,463) pg/ml. Plasma ADTRP outperformed the other three inflammatory biomarkers (TNF-α, IL-6 and hs-CRP) for CAD (Area under ROC curve: 0.67, Odds ratio (OR): 0.907). Our study has shown for the first time that ADTRP is present in circulation, and that plasma ADTRP may be a novel independent biomarker for CAD.

## Introduction

Coronary artery disease (CAD) is one of the leading causes of morbidity and mortality worldwide [1]. According to the World Health Organization (WHO), an estimated 7.4 million global deaths in 2015 was due to CAD [2]. In Singapore, CAD contributes to approximately 17% of deaths per year [3]. CAD is a complex multi-factorial disease caused by the interplay between multiple environmental and genetic factors. Several environmental factors including alcohol consumption, smoking and physical inactivity are identified as risk factors of CAD [4]. The heritability of CAD is estimated to be 50–60% [5] and many candidate genes for CAD have been identified [6]. In addition, genome-wide association studies (GWAS) have continued to identify novel CAD susceptibility genes [7].

A GWAS for CAD conducted in the Chinese Han population had identified a variant, rs6903956, within intron 1 of the C6orf105 gene (also known as the androgen dependent tissue factor pathway inhibitor regulating protein or ADTRP) on chromosome 6p24.1. This variant was shown to be significantly associated with susceptibility to CAD [8]. In addition, its risk allele A was associated with decreased ADTRP mRNA expression, suggesting that low level of ADTRP may confer increased CAD susceptibility [8]. Our group has validated the finding by Wang et al. [8] and found that rs6903956 was also associated with higher CAD risk in our Singaporean Chinese Han population [9].

ADTRP has been reported to regulate mRNA expression of tissue factor pathway inhibitor (TFPI) gene in endothelial cells in an androgen-dependent manner [10]. TFPI is a key natural inhibitor of coagulation as it inhibits the downstream production of thrombin from prothrombin in platelets and prevents thrombosis [11], which may subsequently lead to a lower risk of coronary atherosclerosis. Low serum androgen levels are also associated with increased risk of atherosclerosis and cardiovascular diseases [12]. Androgen is shown to upregulate ADTRP expression and subsequently increasing TFPI expression that exert anti-coagulant effects [10]. Luo et al. suggested that androgen may play a protective role against CAD by inhibiting atherosclerotic processes via direct activation of ADTRP transcription [13].

Other than the evidence that showed association between ADTRP mRNA expression and CAD [8], the protein has not been reported to be present in circulation. The objectives of the present study were: (1) to determine and compare the circulating levels of ADTRP between CAD patients and controls, (2) to test and compare the performance of plasma ADTRP with other existing inflammatory biomarkers in predicting the development of CAD.

## Materials and methods

### Study subjects

The study sample consists of 362 CAD patients and 150 CAD controls who underwent coronary angiography at a local hospital in Singapore. CAD patients were angiographically ascertained to have at least 50% stenosis in at least one of the major coronary arteries: left main (LM) artery, left anterior descending (LAD) artery, left circumflex (LCX) artery, and right coronary artery (RCA). CAD controls were angiographically verified to have less than 30% stenosis, defined as non-significant lesion in all major arteries (LM, LAD, LCX and RCA). Severe CAD cases in this study were defined as patients with myocardial infarction (CAD+MI+) while less severe CAD cases were those without myocardial infarction (CAD+MI-).

The study also included 83 healthy adults who participated in an obesity genetics study conducted at the same hospital. These healthy subjects had no known clinical or medical conditions. Written informed consent was obtained from all study participants and this study was approved by the Domain Specific Review Board (DSRB) of National Healthcare Group, Singapore (DSRB reference no: 2016/00913).

### Measurement of plasma ADTRP levels

Plasma ADTRP was measured using enzyme-linked immunosorbent assay (ELISA) kits for human ADTRP (Lifespan Biosciences Inc., LS-F17830). HUVEC lysates, HUVEC supernatant and recombinant human ADTRP (MyBiosource) were included as positive controls, while PBS was included as negative control in the assays. The manufacturer (Lifespan Biosciences Inc.) reported an inter-assay of <10.20%, and intra-assay of <8.10%, while we reported an inter-assay of 8.74%, and intra-assay of 7.90%. All assays were carried out in accordance with the manufacturers’ protocol and each plasma sample was measured in duplicates.

### Measurement of plasma TNF-α, IL-6 and hs-CRP levels

Plasma tumor necrosis factor-alpha (TNF-α) and interleukin-6 (IL-6) were measured using ELISA kits for human TNF-α (ThermoFisher Scientific) and human IL-6 (Abcam). Plasma high sensitivity-C reactive protein (hs-CRP) levels were measured by the hs-CRP immunoturbidimetric assay on a Beckman Coulter Olympus AU5800 analyzer. All assays were carried out in accordance with the manufacturers’ protocol and each plasma sample was measured in duplicates.

### Statistical analysis

Differences in clinical characteristics between the different groups were analyzed by ANOVA for continuous parameters and Chi-square for categorical parameters, and data were presented as mean ± standard deviation (mean ± SD) or proportion (%). Differences in plasma ADTRP and cytokine levels between the different groups were compared using quantile regression with adjustment for age, gender, ethnicity and BMI, and data were presented as median (interquartile range). Partial Pearson’s correlation was used to examine the correlation between plasma ADTRP and plasma cytokines with adjustment for confounding effects of age, gender, ethnicity and BMI. The association between the various risk factors and CAD were determined by logistic regression. Receiver operating characteristic (ROC) analysis was used to determine the sensitivity and specificity of plasma ADTRP, TNF-α, IL-6 and hs-CRP levels in predicting CAD outcome. Statistical significance for all two-tailed tests was set at *p* < 0.05, and analyses were carried out using IBM SPSS Statistics Version 25 and STATA version 16.

## Results

### Characteristics of study subjects

Our study subjects included 362 CAD patients, 150 CAD controls and 83 healthy adults. CAD patients were significantly older as compared to CAD controls and healthy adults (CAD: ages 57.37 ± 8.56 years; CAD controls: ages 52.05 ± 9.39 years; healthy adults: ages 43.84 ± 6.17 years; p<0.0005). There were also significant differences in the proportion of gender (*p*<0.0005), ethnicity (*p* = 0.002) and in body mass index (BMI) levels (*p* = 0.001) between healthy adults, CAD controls and CAD patients (Table 1).

**Table 1:** Characteristics of study subjects.

	CAD patients	CAD controls	Healthy adults	*p*-value
**Sample size (n)**	362	150	83	-
**Age (years)**	57.37 ± 8.56	52.05 ± 9.39	43.84 ± 6.17	<0.0005*
**Gender (Men/Women) (%)**	86.5 / 13.5	92.0 / 8.0	27.7 / 72.3	<0.0005*
**Ethnicity (Chinese/Malays/Indians) (%)**	56.9 / 27.3 / 15.7	67.3 / 16.7 / 16.0	51.8 / 39.8 / 8.4	0.002*
**BMI (kg/m²)**	26.73 ± 4.21	27.10 ± 5.78	29.04 ± 5.52	0.001*

Data presented as mean ± SD or proportion (%). ANOVA was used to analyze difference in age and BMI between groups. Chi-square was performed to determine differences in proportion of gender and ethnicity between groups. Asterisk (*) denotes significant difference of p < 0.05 between groups.

### Plasma ADTRP, TNF-α, IL-6 and hs-CRP levels in CAD patients and controls

**Fig 1:**
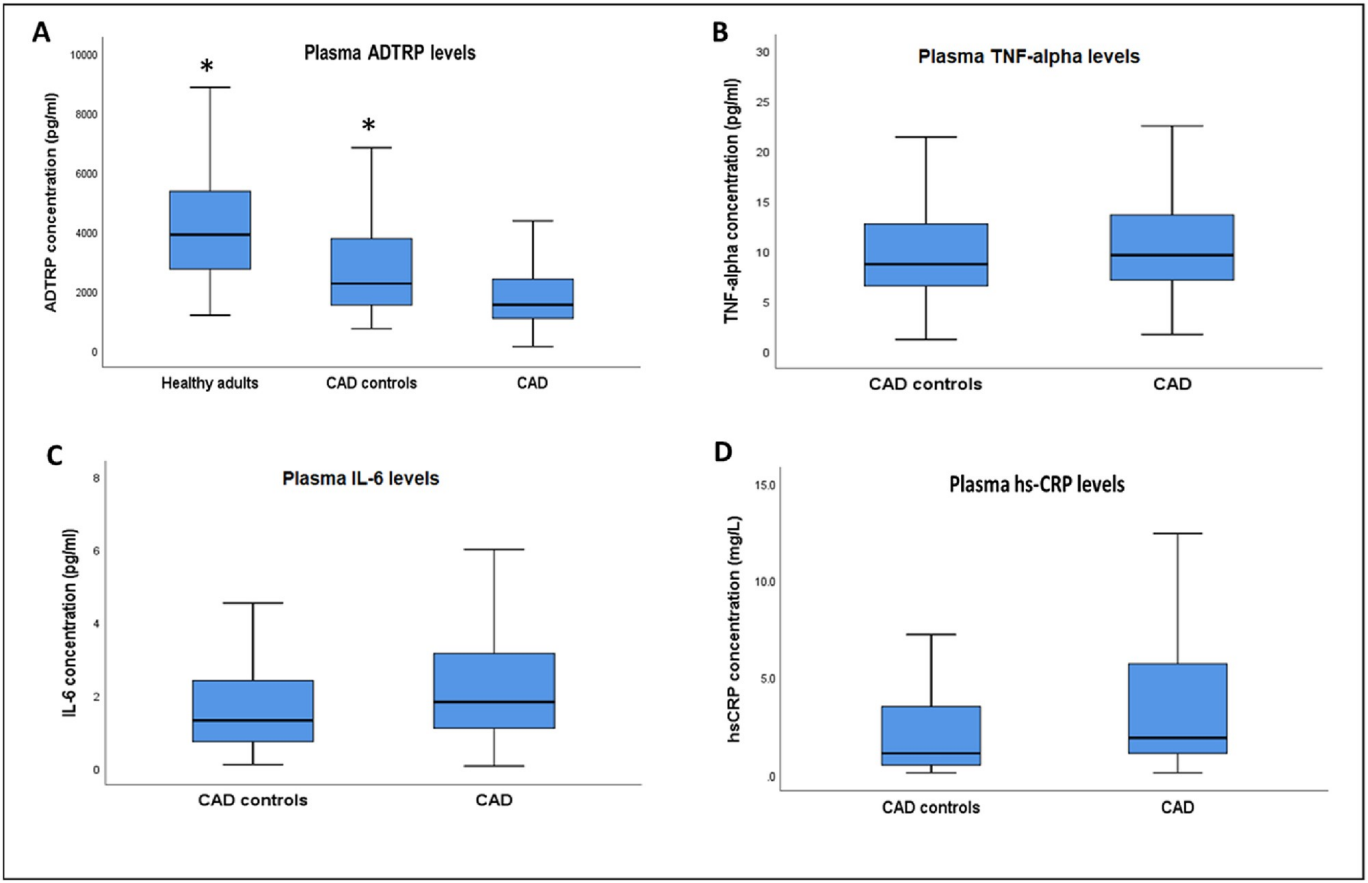
Plasma ADTRP and cytokine levels between CAD patients (n = 362), CAD controls (n = 150) and healthy adults (n = 83). A: Plasma ADTRP, B: Plasma TNF-α, C: Plasma IL-6 and D: Plasma hs-CRP levels. Data are presented as median (interquartile range) and asterisk (*) denotes significant difference of *p*<0.05 as compared to CAD patients. The *p*-values were adjusted for age, gender, ethnicity and BMI using quantile regression.

CAD patients were found to have significantly lower plasma ADTRP levels 1,545 (1,087–2,408) pg/ml as compared to CAD controls 2,259 (1,533–3,778) pg/ml and healthy adults 3,904 (2,732–5,463) pg/ml (Fig 1a). However, there were no significant differences in plasma TNF-α (9.61 (7.09–13.71) pg/ml vs. 8.70 (6.53–12.79) pg/ml), IL-6 (1.82 (1.11–3.16) pg/ml vs. 1.32 (0.74–2.41) pg/ml) and hs-CRP (1.90 (1.05–5.75) mg/L vs. 1.10 (0.5–3.65) mg/L) levels between CAD patients and CAD controls (Fig 1b–1d).

The 362 CAD patients were stratified into whether they had (CAD+MI+, n = 182) or did not have (CAD+MI-, n = 180) a history of myocardial infarction (MI) (S1 Table). There was no significant difference in plasma ADTRP levels between CAD+MI- and CAD+MI+ patients (S1 Fig).

There were also no significant differences in plasma ADTRP levels between genders, ethnic groups, and between smokers and non-smokers (S2 Fig).

### Comparison of plasma ADTRP, TNF-α, IL-6 and hs-CRP’s performance as inflammatory biomarkers for CAD

**Fig 2:**
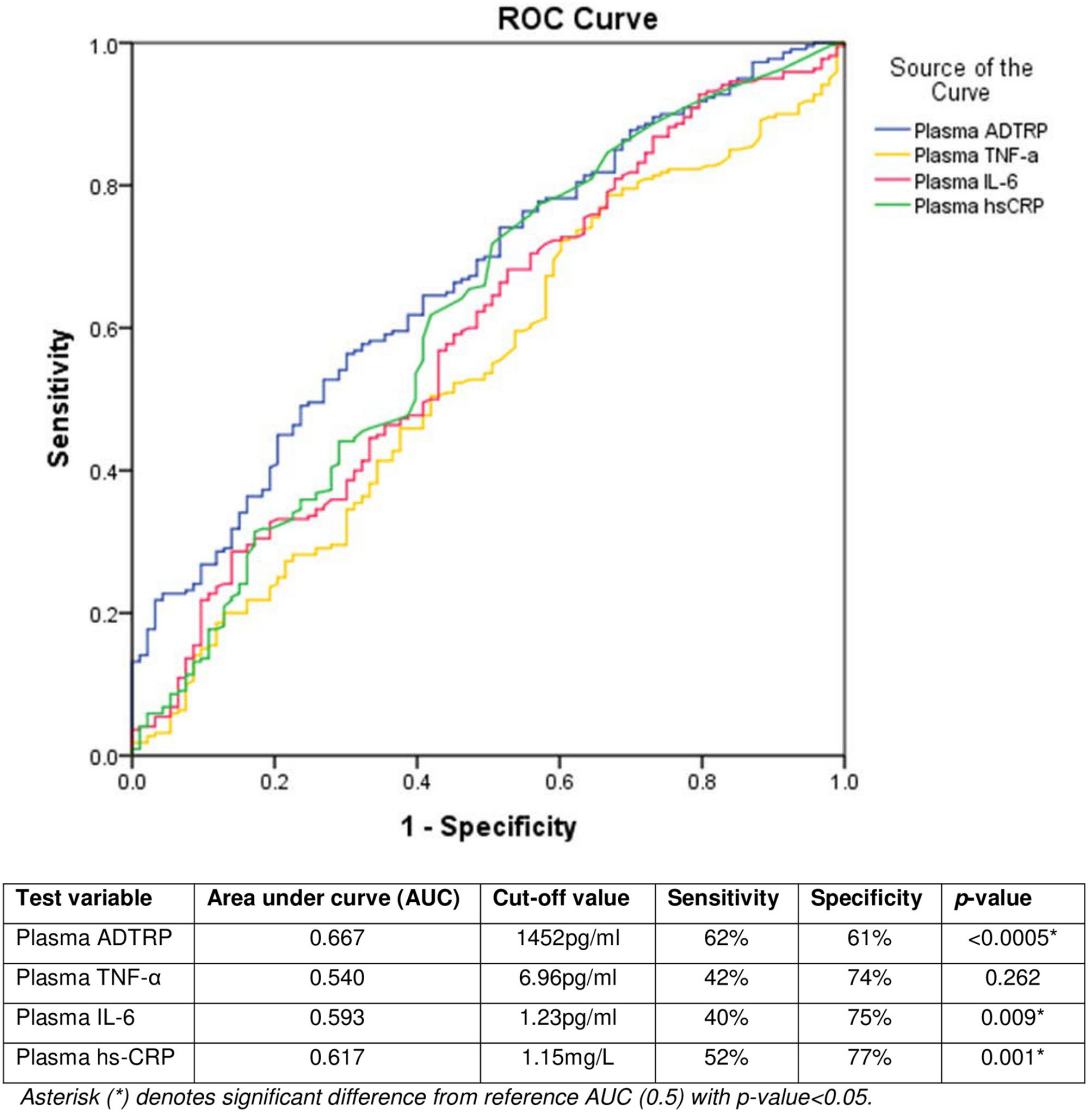
ROC analysis of plasma ADTRP and cytokine levels in predicting CAD (n = 512). Blue line: Plasma ADTRP, Yellow line: Plasma TNF-α, Pink line: Plasma IL-6 and Green line: Plasma hs-CRP levels are shown on the ROC curve. As plasma ADTRP is inversely proportional to CAD risk, its values were multiplied by -1 to enable direct comparison with the curves of the other inflammatory biomarkers.

ROC analysis showed that plasma ADTRP is the most discriminant biomarker for CAD as compared to plasma TNF-α, IL-6 and hs-CRP (Fig 2). The area under the ROC curve for plasma ADTRP level in predicting CAD is 0.667, with sensitivity of 62% and specificity of 61% at a cut-off concentration of 1,452 pg/ml.

Logistic regression analysis showed that plasma ADTRP (OR: 0.907, 95%CI: 0.869–0.945, *p*<0.0005), age (OR: 1.075, 95% CI: 1.037–1.114, *p*<0.0005) and smoking (OR: 2.075, 95% CI: 1.142–3.771, *p* = 0.017) were significant risk predictors of CAD in this study (Table 2). Clearly, the association of ADTRP with CAD is independent of the three other inflammatory biomarkers included in our study and other significant confounding risk factors such as age and smoking.

**Table 2:** Multivariate logistic regression for association of biomarkers and risk factors with CAD.

Variables	CAD		
	Odds Ratio (OR)	95% CI	*p*-value
**Plasma ADTRP (pg/ml)**	0.907	0.869–0.945	<0.0005*
**Plasma TNF-α (pg/ml)**	0.983	0.924–1.045	0.580
**Plasma IL-6 (pg/ml)**	1.101	0.973–1.246	0.126
**Plasma hs-CRP (mg/L)**	0.994	0.966–1.023	0.682
**Age (years)**	1.075	1.037–1.114	<0.0005*
**BMI (kg/m**^**2**^**)**	1.038	0.977–1.104	0.229
**Gender (Women)**	1.834	0.765–4.393	0.174
**Ethnicity (Malays)**	1.944	0.966–3.910	0.062
**Ethnicity (Indians)**	1.162	0.515–2.619	0.718
**Smoking**	2.075	1.142–3.771	0.017*

Asterisk (*) denotes a significant p-value of <0.05.

### Correlation between plasma ADTRP and plasma cytokines in CAD patients and CAD controls

Plasma ADTRP was positively and significantly, though weakly correlated with plasma IL-6 (r = 0.282, *p*<0.0005) and plasma hs-CRP (r = 0.167, *p* = 0.016) (Table 3). The significant correlations were driven mainly by the CAD cases.

**Table 3:** Pearson’s correlation coefficients between plasma ADTRP and plasma cytokines in CAD patients and CAD controls.

	CAD controls and CAD patients		CAD controls		CAD patients	
	Correlation	*p*-value	Correlation	*p*-value	Correlation	*p*-value
**Plasma TNF-α (pg/ml)**	-0.010	0.863	-0.134	0.219	0.054	0.434
**Plasma IL-6 (pg/ml)**	**0.193**	0.001*	0.036	0.742	**0.282**	<0.0005*
**Plasma hs-CRP (mg/L)**	0.108	0.061	0.038	0.729	**0.167**	0.016*

Partial correlation was used for adjustment of age, gender, ethnicity and BMI. Asterisk (*) denotes a significant p-value of <0.05.

## Discussion and conclusion

CAD remains the most prevalent cause of death worldwide [14]. Despite recent advances in diagnosis, treatment and prognosis of cardiovascular diseases, there is still a need to identify novel biomarkers for screening, diagnosis and prognosis. Numerous inflammatory biomarkers for CAD such as CRP, IL-1, IL-6, IL-8 and monocyte chemoattractant protein-1 (MCP-1) have been studied [15–18]. However, many of them were shown to be weak biomarkers and reports on their association with CAD have been inconsistent [19–23].

ADTRP is a novel protein that was reported to be involved in regulating the expression of TFPI in endothelial cells in an androgen-dependent manner. It was postulated to be involved in the pathogenesis of CAD due to its role in regulating TFPI-dependent anti-coagulant mechanism [10]. Chinetti-Gbaguidi et al. showed that ADTRP was also expressed in human macrophages and atherosclerotic lesions, and its expression was upregulated in a peroxisome proliferator-activated receptor-γ (PPAR-γ) dependent-manner [24]. In addition, the ADTRP gene expression in peripheral blood mononuclear cells (PBMCs) of lean subjects was found to be higher than obese diabetic subjects, suggesting that ADTRP may be associated with inflammation in the obese diabetic phenotype [24]. These findings led us to postulate that ADTRP might be involved in the inflammatory processes that contribute to the pathophysiology of atherosclerosis. As such, we measured plasma levels of ADTRP along with known inflammatory biomarkers for CAD: TNF-α, IL-6 and hs-CRP in our study participants comprising CAD patients, CAD controls and healthy adults.

Since the identification of the association of ADTRP genetic variant and its gene expression with CAD, ADTRP has never been directly detected in its protein form. In this study, we have shown that we were able to quantitatively measure plasma ADTRP levels for the first time using the well-established technique of ELISA. We were able to detect ADTRP levels in human umbilical vein endothelial cell (HUVEC) lysates, HUVEC culture supernatant and recombinant human ADTRP using the ELISA kit. Phosphate-buffered saline (PBS) was included as negative control and it was not detectable by the assay.

We observed significantly lower levels of plasma ADTRP in CAD patients than CAD controls and healthy adults. This is consistent with a previous study that showed significant association between decreased ADTRP mRNA expression and CAD susceptibility [8]. The reduced levels of plasma ADTRP in CAD patients may reflect the loss of protection against coagulation. CAD patients had higher levels of plasma TNF-α, IL-6 and hs-CRP than CAD controls but the differences were not statistically significant and this may be attributed to the small sample size.

Consistent with previous studies that reported higher CRP and IL-6 levels in CAD patients with MI as compared to CAD patients without MI [23, 25, 26], we also observed higher plasma IL-6 and plasma hs-CRP levels in CAD+MI+ patients as compared to CAD+MI- patients and CAD controls. In addition, plasma ADTRP levels were found to be significantly lower in CAD+MI- and CAD+MI+ patients as compared to CAD controls. Our results suggest that plasma ADTRP may be a novel biomarker for CAD. We observed no significant difference in plasma ADTRP between genders, ethnic groups and between smokers and non-smokers.

Inflammatory biomarkers are found to be predictive of CAD risk [27]. ROC analysis in our study revealed that plasma ADTRP is a better biomarker for CAD than the other three inflammatory biomarkers i.e. TNF-α, IL-6 and hs-CRP that were included in this study. Multivariate logistic regression also showed that plasma ADTRP is a protective marker, with every 100 pg/ml increase in ADTRP corresponding to a reduction of CAD risk by 9.3%. Our data demonstrated that plasma ADTRP level may be a good candidate for predicting development of CAD. However, a larger prospective longitudinal study would be required to assess the usefulness of plasma ADTRP level as a prognostic and predictive biomarker of CAD.

Notwithstanding the notable findings from this study, we recognized that there are also several limitations. Firstly, although our results have demonstrated that plasma ADTRP may be a good candidate in predicting development of CAD, this study was case-control in design and hence was not able to differentiate cause and effect. A larger prospective study is therefore necessary to eliminate the possibility that ADTRP might be a consequence of having CAD rather than a cause. Nevertheless, the lower level of ADTRP observed in CAD patients as compared to controls suggested a possible role of ADTRP in the pathogenesis of CAD. Secondly, our CAD patients were significantly older than the CAD controls as most of the CAD controls who were admitted for coronary angiography and found to have less than 30% coronary stenosis were younger than the CAD patients. We were hence unable to achieve an ideal match of their ages. However, we had adjusted for the confounding effect of age on plasma ADTRP levels in our analysis. Thirdly, our study had fewer CAD controls (n = 150) than CAD patients (n = 362) as the initial aim of the study was to examine plasma ADTRP levels in three groups, CAD+MI- patients (n = 180), CAD+MI+ patients (n = 182) and CAD controls (n = 150). However, as our results showed no significant difference in plasma ADTRP levels between the CAD+MI- and CAD+MI+ patients, they were combined, as one CAD patient group. We also measured plasma ADTRP in 83 healthy adults, and showed that plasma ADTRP was higher in the healthy subjects, further supporting the association between low ADTRP levels and CAD. Lastly, multiple inflammatory biomarkers are found to be associated with CAD but due to the limited availability of the plasma samples, we only measured the levels of TNF-α, IL-6 and hs-CRP which have been extensively reported to be potential predictive biomarkers for CAD risk [27]. Hence, further studies are required to compare the performance between plasma ADTRP and other biomarkers for CAD.

In conclusion, our study has shown, for the first time, that the novel protein ADTRP is present in circulation and that its levels are significantly lower in CAD patients. Plasma ADTRP may potentially be a novel independent biomarker for CAD.

## Supporting information

S1 Table Characteristics of healthy adults, CAD controls and CAD patients with and without history of MI. Data presented as mean ± SD or proportion (%). ANOVA was used to analyze difference in age and BMI between groups. Chi-square was performed to determine differences in proportion of gender and ethnicity between groups. Asterisk (*) denotes significant difference of p < 0.05 between groups.
(PDF)

S1 Fig Plasma ADTRP and cytokine levels between healthy adults (n = 83), CAD controls (n = 150), CAD patients without history of MI (CAD+MI-) (n = 180) and CAD patients with history of MI (CAD+MI+) (n = 182). A: Plasma ADTRP, B: Plasma TNF-α, C: Plasma IL-6 and D: Plasma hs-CRP levels. Data are presented as median (interquartile range). Asterisk (*) denotes significant difference of *p*<0.05 as compared to healthy adults and # denotes significant difference of p<0.05 as compared to CAD controls. The *p*-values were adjusted for age, gender, ethnicity and BMI using quantile regression.
(PDF)

S2 Fig Plasma ADTRP levels in CAD patients, CAD controls and healthy adults. A: Plasma ADTRP levels between different genders, men (n = 474) and women (n = 121), B: Plasma ADTRP levels between different ethnic groups, Chinese (n = 350), Malays (n = 157) and Indians (n = 88), C: Plasma ADTRP levels between non-smokers (n = 208) and smokers (n = 304). Data are presented as median (interquartile range). The *p*-values were adjusted for age, gender, ethnicity, BMI and CAD status using quantile regression.
(PDF)

## Abbreviations

ADTRP: Androgen dependent tissue factor pathway inhibitor regulating protein
AUC: Area under curve
BMI: Body mass index
CAD: Coronary Artery Disease
CRP: C Reactive Protein
ELISA: Enzyme-linked immunosorbent assay
GLM: General linear model
GWAS: Genome-wide association studies
hs-CRP: High sensitivity-C Reactive Protein
HUVEC: Human umbilical vein endothelial cell
IL: Interleukin
LAD: Left anterior descending
LCX: Left circumflex
LM: Left main
MCP-1: Monocyte chemoattractant protein-1
MI: Myocardial Infarction
OR: Odds Ratio
PBS: Phosphate-buffered saline
RCA: Right coronary artery
ROC: Receiver operating characteristic
TFPI: Tissue factor pathway inhibitor
TNF-α: Tumor necrosis factor-alpha
