# Supplementary material for: Detection of ADTRP in circulation and its role as a novel biomarker for coronary artery disease
Source: PLoS One. 2020 Aug 13;15(8):e0237074. doi: 10.1371/journal.pone.0237074 (PMC7425853; doi:10.1371/journal.pone.0237074)
Supplement: S1 Table — Data presented as mean ± SD or proportion (%). ANOVA was used to analyze difference in age and BMI between groups. Chi-square was performed to determine differences in proportion of gender and ethnicity between groups. Asterisk (*) denotes significant difference of p < 0.05 between groups. (PDF) [file pone.0237074.s001.pdf]

	Healthy adults	CAD Controls	CAD+MI-	CAD+MI+	P-value
**Sample size (n)**	83	150	180	182	-
**Age (years)**	43.84 ± 6.17	52.05 ± 9.39	57.87 ± 8.05	56.89 ± 9.03	**<0.0005***
**Gender (Men/Women) (%)**	27.7/72.3	92.0/8.0	88.3/11.7	84.6/15.4	**<0.0005***
**Ethnicity (Chinese/Malays/Indians) (%)**	51.8/39.8/8.4	67.3/16.7/16.0	65.0/22.2/12.8	48.9/32.4/18.7	**<0.0005***
**BMI (kg/m²)**	29.04 ± 5.52	27.10 ± 5.78	26.66 ± 4.13	26.79 ± 4.30	**0.002***
