# Supplementary figures and images for: Detection of ADTRP in circulation and its role as a novel biomarker for coronary artery disease
Source: PLoS One. 2020 Aug 13;15(8):e0237074. doi: 10.1371/journal.pone.0237074 (PMC7425853; doi:10.1371/journal.pone.0237074)

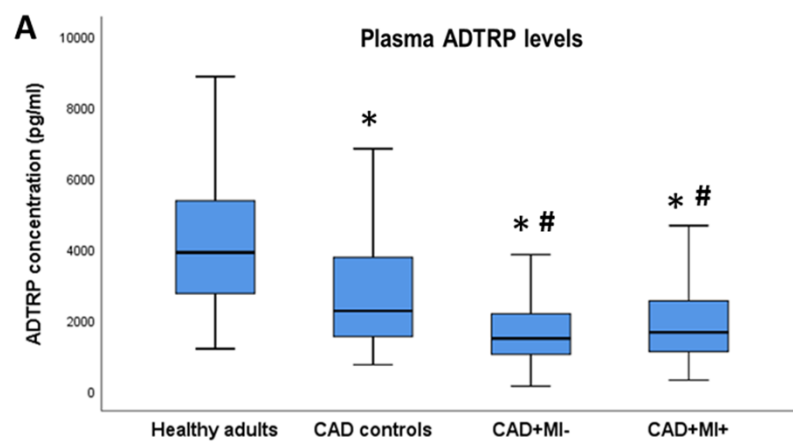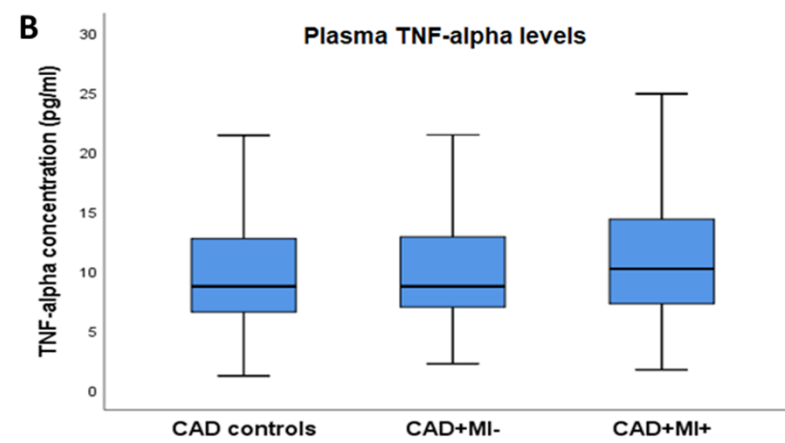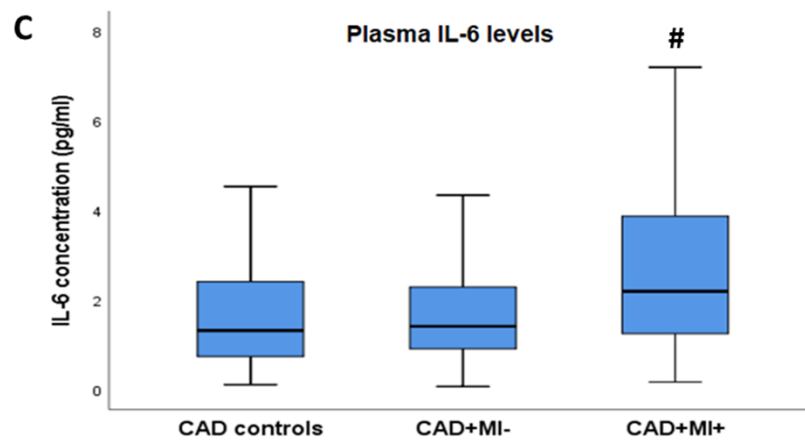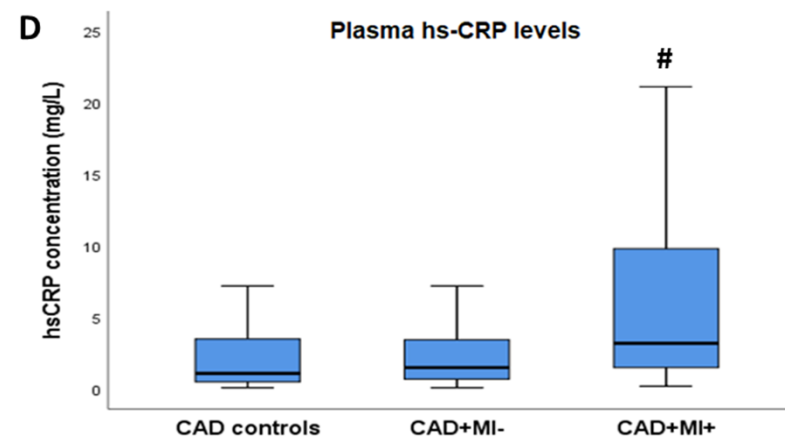

Supplement: S1 Fig — A: Plasma ADTRP, B: Plasma TNF-α, C: Plasma IL-6 and D: Plasma hs-CRP levels. Data are presented as median (interquartile range). Asterisk (*) denotes significant difference of p<0.05 as compared to healthy adults and # denotes significant difference of p<0.05 as compared to CAD controls. The p-values were adjusted for age, gender, ethnicity and BMI using quantile regression. (PDF) [file pone.0237074.s002.pdf]

## Plasma ADTRP levels

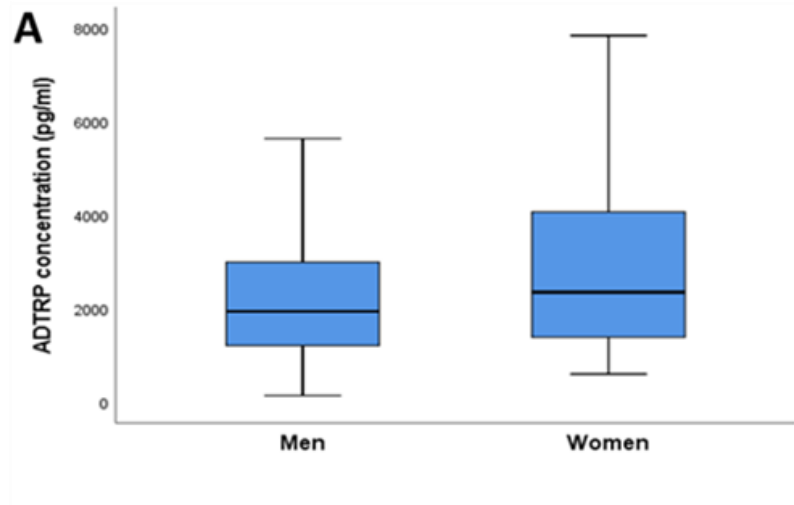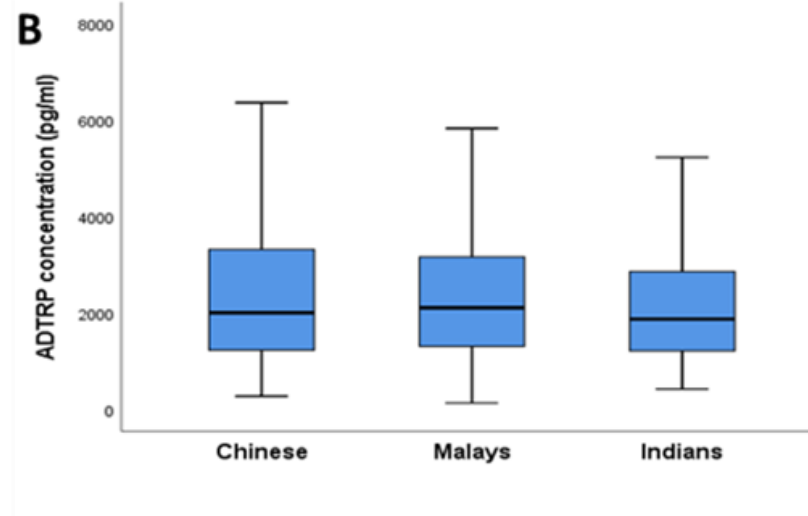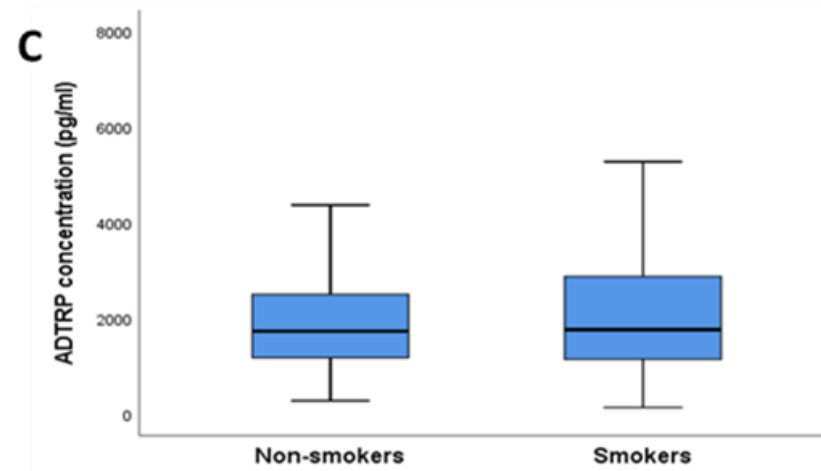

Supplement: S2 Fig — A: Plasma ADTRP levels between different genders, men (n = 474) and women (n = 121), B: Plasma ADTRP levels between different ethnic groups, Chinese (n = 350), Malays (n = 157) and Indians (n = 88), C: Plasma ADTRP levels between non-smokers (n = 208) and smokers (n = 304). Data are presented as median (interquartile range). The p-values were adjusted for age, gender, ethnicity, BMI and CAD status using quantile regression. (PDF) [file pone.0237074.s003.pdf]
